# Intratracheal transplantation of mesenchymal stem cells attenuates hyperoxia-induced lung injury by down-regulating, but not direct inhibiting formyl peptide receptor 1 in the newborn mice

**DOI:** 10.1371/journal.pone.0206311

**Published:** 2018-10-24

**Authors:** Young Eun Kim, Won Soon Park, So Yoon Ahn, Dong Kyung Sung, Yun Sil Chang

**Affiliations:** 1 Department of Health Sciences and Technology, Samsung Advanced Institute for Health Sciences and Technology (SAIHST), Sungkyunkwan University, Seoul, Korea; 2 Stem Cell and Regenerative Medicine Institute, Samsung Medical Center, Seoul, Korea; 3 Department of Pediatrics, Samsung Medical Center, Seoul, Korea; 4 Samsung Biomedical Research Institute, Sungkyunkwan University School of Medicine, Seoul, Korea; Center of Pediatrics, GERMANY

## Abstract

Formyl peptide receptor 1 (FPR1) has been shown to be a key regulator of inflammation. However, its role in bronchopulmonary dysplasia (BPD) has not been delineated yet. We investigated whether FPR1 plays a pivotal role in regulating lung inflammation and injuries, and whether intratracheally transplanted mesenchymal stem cells (MSCs) attenuate hyperoxic lung inflammation and injuries by down-regulating FPR1. Newborn wild type (WT) or FPR1 knockout (FPR1^-/-^) C57/BL6 mice were randomly exposed to 80% oxygen or room air for 14 days. At postnatal day (P) 5, 2×10^5^ MSCs were intratracheally transplanted. At P14, mice were sacrificed for histopathological and morphometric analyses. Hyperoxia significantly increased lung neutrophils, macrophages, and TUNEL-positive cells, while impairing alveolarization and angiogenesis, along with a significant increase in FPR1 mRNA levels in WT mice. The hyperoxia-induced lung inflammation and lung injuries were significantly attenuated, with the reduced mRNA level of FPR1, in WT mice with MSC transplantation and in FPR1^-/-^ mice, irrespective of MSCs transplantation. However, only MSC transplantation, but not the FPR1 knockout, significantly attenuated the hyperoxia-induced increase in TUNEL-positive cells. Our findings indicate that FPR1 play a critical role in regulating lung inflammation and injuries in BPD, and MSCs attenuate hyperoxic lung inflammation and injuries, but not apoptosis, with down regulating, but not direct inhibiting FPR1.

## Introduction

Bronchopulmonary dysplasia (BPD), a chronic pulmonary disease occurring in premature infants receiving prolonged mechanical ventilation and oxygen supplementation, remains a major cause of mortality and long-term respiratory and neurodevelopmental morbidities with few effective treatments [1, 2]. Although BPD has a multifactorial etiology, inflammation is believed to play a key role in the lung injury process leading to the development of histopathological characteristics of BPD including impaired alveolarization and increased fibrosis [3, 4].

We recently reported the therapeutic efficacy of human umbilical cord blood (UCB) derived mesenchymal stem cells (MSCs) in protecting against hyperoxic lung injuries in newborn rats [5], the safety and feasibility of this cell therapy in preterm infants at risk for developing BPD in a phase I clinical trial [6], and a follow-up of these infants for up to 2 years of the corrected age [7]. The transplanted MSCs exert their therapeutic effects by sensing the microenvironment of the host tissue injury site and then secreting various paracrine factors that have several reparative functions, including anti-apoptotic, anti-inflammatory, anti-oxidative, anti-fibrotic, and/or antibacterial effects in response to the environmental cues to enhance regeneration of the damaged tissue [8, 9]. The pleiotropic protective effects of MSC transplantation suggest that stem cell therapy could be the next breakthrough for treating currently intractable and devastating neonatal disorder with complex multifactorial etiologies, such as BPD. However, a better understanding of the paracrine protective molecular mechanism of action is essential for its future application in clinical care.

Formyl peptide receptor (FPR) 1, a well-conserved G protein receptor, is a potential key receptor involved in the acute antimicrobial and inflammatory process with the capacity to sense and respond to unique bacterial and host-derived mitochondrial DNA and formylated peptides, stimulating neutrophil chemotaxis, degranulation, production of reactive oxygen species, and cytokine release [10–13]. In acute respiratory distress syndrome (ARDS), elevated mitochondrial formylated peptides induced sterile acute lung inflammation and injury through FPR1 signaling, thereby suggesting a potential new therapeutic target in ARDS [14]. In our previous study, we performed microarray analyses of MSC transplantation for BPD in newborn rats [5], and we observed upregulation of FPR1 in BPD, and downregulation of FPR1 with MSC transplantation (unpublished data). However, the precise role of FPR1 in BPD and stem cell therapy remains to be elucidated.

In this study, we investigated the role of FPR1 signaling in the pathogenesis of hyperoxia-induced lung inflammation and the ensuing impaired alveolarization and angiogeneisis in mice, and the influences on FPR1 expression with MSC transplantation in this context. To this end, we assessed the extent of hyperoxia induced lung inflammation and injuries, with respect to the expression of lung FPR1, under hyperoxic and MSC transplantation conditions in wild type and FPR1 knockout (FPR1^-/-^) mice.

## Materials and methods

### Mesenchymal stem cells

As previously described [15], umbilical cord blood (UCB) was collected from umbilical veins after neonatal delivery with maternal informed consent. UCB harvests were processed within 24 h of collection. UCB was separated by isolating mononuclear cells with Ficoll-Hypaque solution (density, 1.077g/cm3; Sigma, St. Louis, MO, USA). The separated mononuclear cells were washed, suspended in Minimum Essential Medium a modification (MEM a; Gibco, Carlsbad, CA, USA) supplemented with 10% fetal bovine serum (FBS; Gibco), and seeded at a concentration of 5 x 10^5^ cells/cm^2^. Cultures were maintained at 37°C in a humidified atmosphere (21% O_2_) containing 5% CO_2_ with a change of culture medium twice a week. One batch of cells was used to inject all pups. As described previously [16], human UCB derived MSCs from a single donor at passage 6 manufactured and also used for various preclinical studies of neonatal intractable disorders including BPD [17], IVH [18] and HIE [19] were obtained from Medipost Co., Ltd. (Seoul, Korea).

### Animal model

The animal experimental protocol was reviewed and approved by the Institutional Animal Care and Use Committee of Samsung Biomedical Research Institute (Seoul, Korea). The experimental procedures were also performed in accordance with the National Institutes of Health Guidelines for Laboratory Animal Care, in addition to our institutional guidelines. All animal procedures were performed in an AAA-LAC-accredited specific pathogen free facility. Timed pregnant wild type (WT) C57/BL6 mice (Orient Co. Ltd, Gapyoung, Korea) and C57/BL6 FPR1 knockout (FPR1^-/-^) mice were a kind gift from Professor Jae Ho Kim, Pusan National University, Yangsan, Korea. To genetically inactivate FPR1, a 150-bp ORF fragment of Fpr1 was replaced with a neomycin resistance cassette, which used to select for targeted events [10]. The deleted region is from the first extracellular loop to the fourth transmembrane segment predicted from the FPR sequence (codons 101–150), where is crucial for FPR1 activation. Then, the mutant, FPR1^-/-^, mice was confirmed by genotyping using PCR, following instruction of the laboratory which developed the mutant mice (S2 Fig) [10]. Dam mice were maintained in an alternating 12-hour light/dark cycle with constant room humidity and temperature. Newborn WT and FPR1^-/-^ mice were reared with their dams in individual cages with free access to water and laboratory chow. We assessed and monitored the condition of mouse pups on weekly basis regularly and twice per day in a daily basis especially for the 14 days after hyperoxia exposure. In this study, we used humane endpoint as the earliest indicator in an animal experiment of pain or distress that could be used to avoid or limit pain and distress by taking actions such as humane euthanasia. For humane endpoint, operationally defined scoring system was approved by IACUC. Total scores of with or more than 5 or score 3 in any single category were arbitrarily defined as humane endpoint. Humane endpoints consist of body weight growth (1: slower growth than normal mice, 2: growth arrest, 3: weight loss), responsiveness (1: delayed but appropriate response, 2: delayed and null response, 3: no response), and appearance (1: rough hair coat, 2: porphyrin staining, 3: sustained abnormal posture or dilated pupil). Throughout the experimental period, no mouse pups reached a humane endpoint. Litters from the same mother randomized over different groups. Immediately after birth, in wild type or FPR1^-/-^ mice, mouse pups were randomly divided according to normoxia (N) or hyperoxia (H) into two groups for a total of three groups: normoxia control (NC), hyperoxia control (HC) and hyperoxia with MSCs transplantation (HM). And at postnatal day 5, the hyperoxia group was randomly divided into hyperoxia control (HC) and hyperoxia with MSC transplantation (HM) group. Adult mice have a poor tolerance to 80% hyperoxia for more than 24 hours, so we daily rotated nursing mother mice between litters in the normoxia and hyperxoxia groups to avoid oxygen toxicity. Gender was not considered when the pups were randomized, and all female and male mice were used in this study. With their dams, normoxia groups were raised in room air and hyperoxia groups were in hyperoxic chamber (80% oxygen) from birth until sacrifice at postnatal day (P) 14 for morphologic and biochemical analyses. At P5, 2×10^5^ human UCB- derived MSCs in 20μl of normal saline were transplanted intratracheally to hyperoxic or normxic mice in WT and FPR1^-/-^ mice. For intratracheal administration of MSCs in the very tiny mouse pups weighing approximately 1.8–2.2g, we anesthetized them with an intraperitoneal injection of a ketamine and xylazine mixture (45 mg/kg and 8 mg/kg, respectively), and restrained them on a board at a fixed angle and injected MSCs into the trachea through a 31-gauge needle syringe, as previously reported [16]. After the procedure the animals were allowed to recover from the anesthesia and were returned to their dam. We used 84 animals in total, and 6 to 8 animals per group were used for every read-out in histological and biochemical analysis, respectively. There was no death during the animal experiment.

### Tissue preparation

At P14, under deep pentobarbital anesthesia (60 mg/kg, i.p.), the lungs were harvested immediately following transcardiac perfusion with ice-cold PBS. Tissue preparation for PCR and histology was performed in different mouse pups. For lung morphometry and immunohistochemistry, extracted lung tissues were inflated with PBS at a constant inflation pressure of 20cm H_2_O and then immersion-fixed as previously described [20]. Paraffin blocks of lung tissues were then sliced into 4 μm sections. For biochemical observations, the lungs were snap frozen in liquid nitrogen and stored at −80°C until use.

### FPR1 mRNA expression

FPR1 mRNA level in lung tissue was measured by reverse transcription-PCR (RT-PCR). Total RNA was isolated from homogenized lung tissue using Trizol reagent (Invitrogen, La Jolla, CA, USA) according to the manufacturer’s instruction. From the total RNA, complementary DNA (cDNA) was synthesized using SMARTScribe Reverse Transcriptase (Clontech, Tokyo, Japan) with pd(N)6 random hexamers (Bioneer, Daejeon, Korea), following the manufacturer's instruction. Using 1 microliter of cDNA (250 ng/μl), PCR amplifications of FPR1 and GAPDH, a housekeeping gene were performed by the following conditions: 5 min hot start at 94°C; followed by 32 cycles of 94°C for 30 s, 57°C for 30 s, 72°C for 30 s; and a final extension at 72°C for 5 min (FPR1 forward primer sequence, 5’-CCTTGGCTTTCTTCAACAGC-3’; FPR1 reverse primer sequence, 5’-GCCCGTTCTTTACATTGCAT-3’; GAPDH forward primer sequence, 5’-CGTCCCGTAGACAAAATGGT-3’; GAPDH reverse primer sequence, 5’-TTGATGGCAACAATCTCCAC-3’). The PCR products were resolved and visualized by E-Gel Power Snap Electrophoresis System (Invitrogen, Massachusetts, USA). PCR band intensities of FPR1 and GAPDH were measured using ImageJ software (National Institutes of Health, Bethesda, MD) and used to calculate FPR1/GAPDH ratio.

### Lung morphometry

Paraffin-embedded lung sections (4-μm thick sections) were stained with hematoxylin and eosin. The level of alveolarization was evaluated by mean linear intercept (MLI) [21], mean alveolar volume (MAV) [22] and radial alveolar count (RAC) [23]. A minimum of 6 non-overlapping microscopic fields (×200 magnification; MLI and MAV, ×35 magnification; RAC) from each section were randomly chosen for morphometric measurements.

### Immunohistochemistry

For histological analyses of inflammation, paraffin-embedded lung sections were immunostained with CD68 (1:100; ab31630, Abcam, Cambridge, UK) and myeloperoxidase (MPO) (1:25; ab9535, Abcam) primary antibodies. The number of CD68-positive alveolar macrophages and MPO-positive polymorphonuclear neutrophils were counted on at least six non-overlapping fields at a magnification of 200×. For histological analyses of angiogenesis, the lung sections were immunostained for von Willebrand factor (vWF), a marker for endothelial cells (ready to use; IR527, FLEX, Dako, Glostrup, Denmark). To quantify angiogenesis, light intensities of vWF-positive cells were measured in at least 6 non-overlapping fields at a magnification of 100× using the ImageJ software (National Institutes of Health, Bethesda, MD), and the numbers of vWF-positive vessels were counted. All quantifications of histological analyses were performed in an observer-blinded manner.

### Enzyme-linked immunosorbent assay

Following homogenization and centrifugation of frozen lung tissues, the protein concentration in each supernatant was standardized across all samples. The levels of VEGF were measured using commercial enzyme‐linked immunoabsorbent assays (ELISA) (VEGF‐R&D Systems, Minneapolis, MN, USA)

### Western blot

For biochemical analyses of angiogenesis and apoptosis, CD31 and caspase 9 in lung tissues were detected by western blot. The membranes were blocked and incubated with the primary antibodies of CD31 (1:1000; sc-376764, Santa Cruz Biotechnology, Santa Cruz, CA, USA) and caspase 9 (1:500; sc-7885, Santa Cruz Biotechnology), followed by secondary antibody incubation (1:1,000; DAKO). Level of housekeeping protein, glyceraldehyde-3-phosphate dehydrogenase (GAPDH, 1:1000; sc-25778, Santa Cruz Biotechnology), was measured as loading control. Protein signals were developed by ECL Prime Western blotting detection reagent (GE Healthcare, Piscataway, NJ, USA) and detected by Amersham Imager 600 (GE Healthcare Life Sciences; Pittsburg PA, USA). Detected band intensities were measured using ImageJ software (National Institutes of Health, Bethesda, MD, USA) and the probing protein/GAPDH ratio was calculated from the band intensities.

### TUNEL staining

To observe cell death in lung tissues, TUNEL staining was performed on lung sections using the DeadEnd Fluorometric TUNEL System kit (G3250; Promega, Madison, WI). The TUNEL-stained lung sections were observed at 200× magnification. The number of TUNEL- positive cells was counted in six non-overlapping fields in an observer-blinded manner.

### Statistical analysis

Data were expressed as mean ± SEM. After normal distribution was tested, in power calculation analyses, we used the data for lung aveolarization (mean linear intercept), which is the most important morphometric parameter in hyeroxic lung injury, between wild type of hyperoxia control (n = 8) and FPR1^-/-^ mice of hypeorxia control (n = 6). When we set a significance level (alpha) at 0.05 and a power at 0.8, our group sample sizes of 8 and 6 achieve 81% power. It shows 6 to 8 subjects were enough to the study. For continuous variables, statistical comparison between groups was by one-way analysis of variance (ANOVA) and Tukey’s post hoc analysis. All data were analyzed using SAS 9.4 software (SAS Institute, Cary, NC), and *P* values less than 0.05 were considered statistically significant.

## Results

### FPR1 expression level

**Fig 1:**
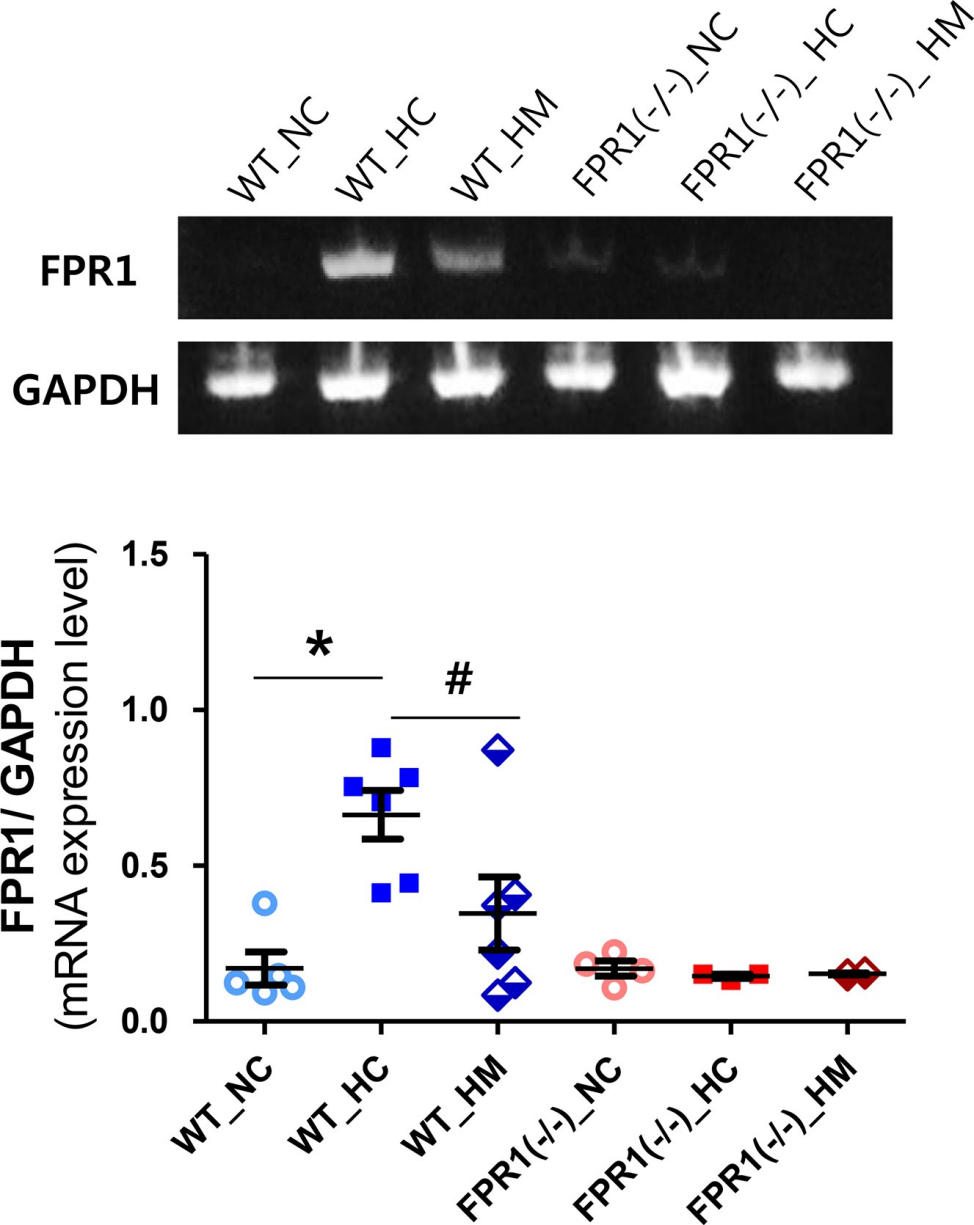
mRNA level of formyl peptide receptor (FPR) 1 in mouse lung tissue. Representative RT-PCR blot of FPR1 (upper panel) and its densitometric histogram, normalized to glyceraldehyde-3-phosphate dehydrogenase (GAPDH), of each group (lower panel). WT-NC, wild type mice of normoxia control; WT-HC, wild type mice of hyperoxia control; WT-HM, wild type mice of hyperoxia with MSCs; FPR1^-/-^ -NC, FPR1 knockout mice of normoxia control; FPR1^-/-^ -HC, FPR1 knockout mice of hyperoxia control; FPR1^-/-^ -HM, FPR1 knockout mice of hyperoxia with MSCs (n = 6, 8, 7, 6, 7 and 6 in WT-NC, WT-HC, WT-HM, FPR1^-/-^ -NC, FPR1^-/-^ -HC and FPR1^-/-^ -HM, respectively) Data are given as mean ± SEM. * P < 0.05 vs. WT-NC. # P < 0.05 vs. WT-HC.

In wild type mouse lungs, the FPR1 mRNA expression level was significantly increased after hyperoxic exposure in the WT-HC group compared to WT-NC group (Fig 1). This hyperoxia-induced increase in FPR1 mRNA level was significantly attenuated after intratracheal transplantation of human UCB-derived MSCs in the WT-HM group. In FPR1^-/-^mice lung, the FPR1 mRNA level was not changed from hyperoxia and UCB-MSC transplantation. In normoxia condition, the intratracheal transplantation of MSCs did not change the FPR1 mRNA expression level in WT-NM and FPR1^-/—^NM groups, compared to WT-NC and FPR1^-/—^NC groups (S1 Fig).

### Lung alveolarization

**Fig 2:**
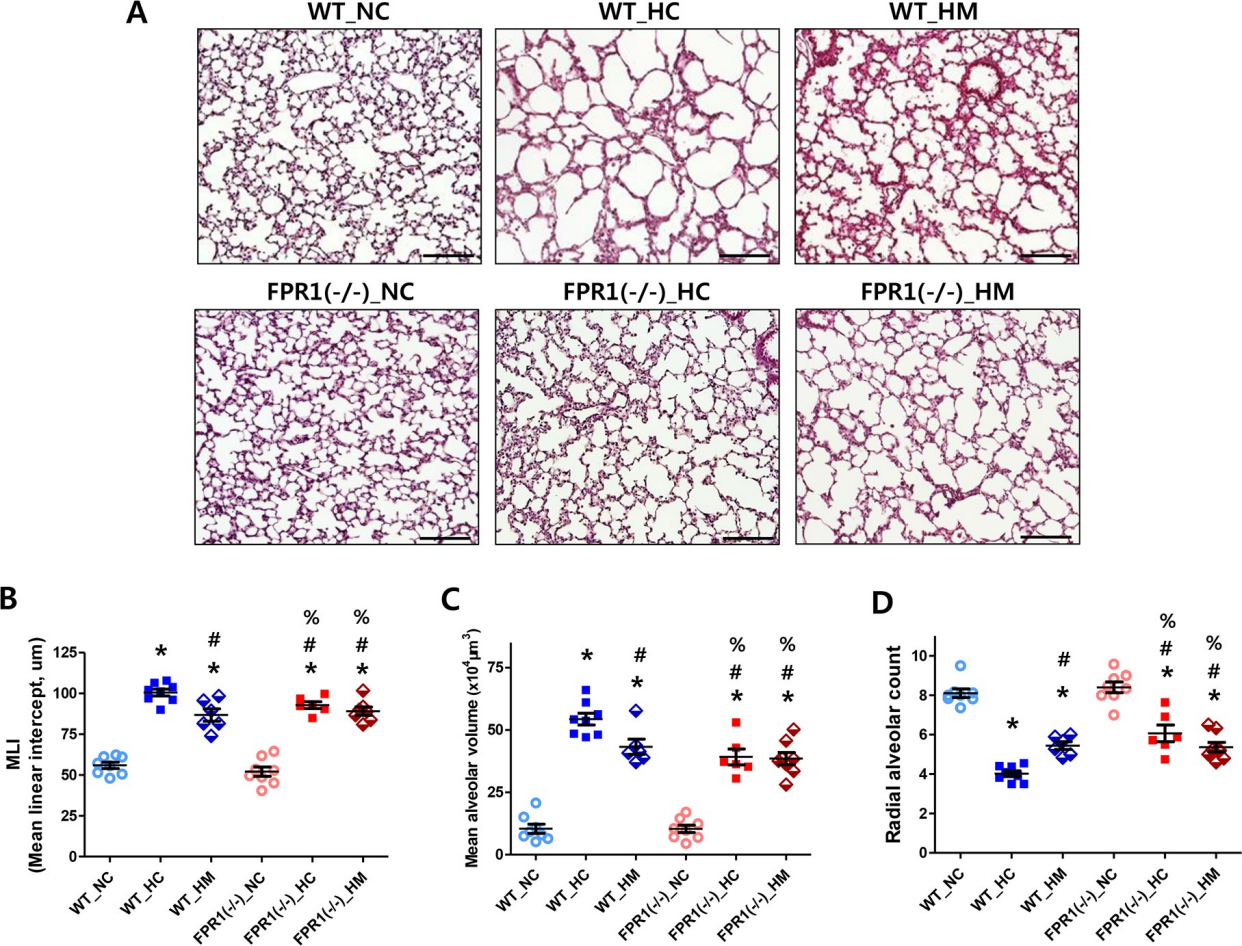
Morphometric evaluation of alveolarization. (A) Representative photomicrographs of hematoxylin and eosin stained lung tissues (left panel, original magnification; ×200, scale bar; 100μm). Degree of alveolarization measured by the mean linear intercept (B) and mean alveolar volume (C) of each group. WT-NC, wild type mice of normoxia control; WT-HC, wild type mice of hyperoxia control; WT-HM, wild type mice of hyperoxia with MSCs; FPR1^-/-^ -NC, FPR1 knockout mice of normoxia control; FPR1^-/-^ -HC, FPR1 knockout mice of hyperoxia control; FPR1^-/-^ -HM, FPR1 knockout mice of hyperoxia with MSCs (n = 8, 8, 6, 8, 6 and 8 in WT-NC, WT-HC, WT-HM, FPR1^-/-^ -NC, FPR1^-/-^ -HC and FPR1^-/-^ -HM, respectively) Data are given as mean ± SEM. * P < 0.05 vs. WT-NC. # P < 0.05 vs. WT-HC, % P < 0.05 vs. FPR1^-/-^ -NC.

Fig 2A demonstrates representative light microscopic photomicrographs showing the histopathological differences in each experimental group. Compared to the small and uniform alveoli in the NC groups, fewer, larger, and heterogenous alveoli were observed in the WT HC group. The hyperoxia induced impaired alveolarization was attenuated with MSC transplantation in the WT-HC group as well as in the FPR1^-/-^ group without the MSC transplantation. Morphometric analyses revealed that MLI and MAV were significantly higher, and RAC was significantly lower in WT-HC group then NC groups (Fig 2B–2D). The hyperoxia-induced morphometric abnormalities were significantly attenuated with MSC transplantation in the WT group as well as in the FPR1^-/-^ group regardless of MSC transplantation.

### Angiogenesis

**Fig 3:**
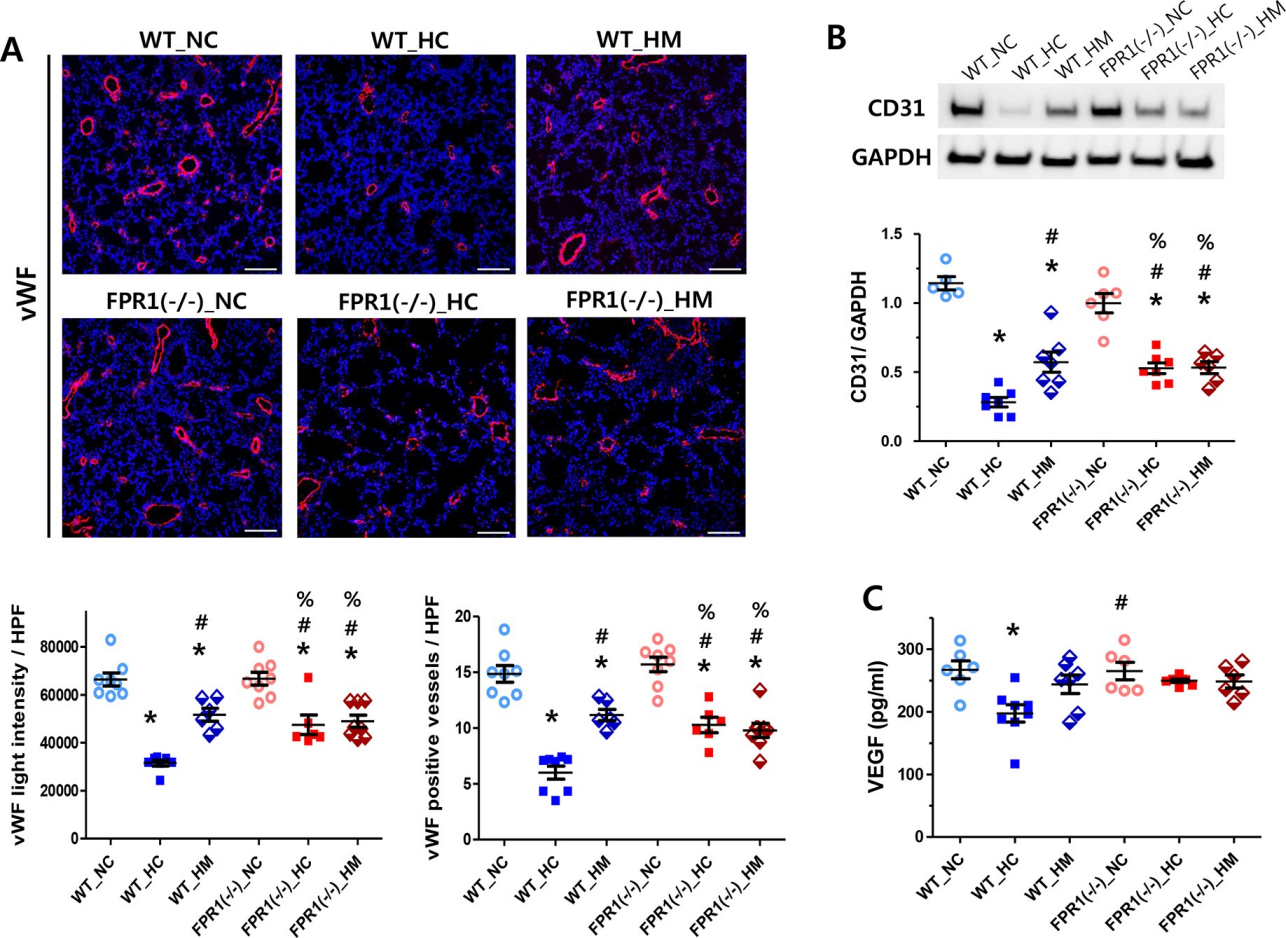
Angiogenesis in lung tissues. (A) Representative confocal images of von willbrand factor (vWF; red) with DAPI (blue) (upper panel, original magnification; ×100, scale bar; 100μm) and the light intensity and the vessel number of vWF-positive cells per high power field of each group (lower panel). (n = 8, 8, 6, 8, 6 and 8 in WT-NC, WT-HC, WT-HM, FPR1^-/-^ -NC, FPR1^-/-^ -HC and FPR1^-/-^ -HM, respectively) (B) Representative western blots of CD31 and glyceraldehyde-3-phos phosphatedehydrogenase (GAPDH; loading control) and densitometric analysis of CD31 levels normalized to GAPDH (n = 6, 8, 7, 6, 7 and 6 in WT-NC, WT-HC, WT-HM, FPR1^-/-^ -NC, FPR1^-/-^ -HC and FPR1^-/-^ -HM, respectively) (C) Level of vascular endothelial growth factor (VEGF) in lung tissue (n = 6, 8, 7, 6, 7 and 6 in WT-NC, WT-HC, WT-HM, FPR1^-/-^ -NC, FPR1^-/-^ -HC and FPR1^-/-^ -HM, respectively). WT-NC, wild type mice of normoxia control; WT-HC, wild type mice of hyperoxia control; WT-HM, wild type mice of hyperoxia with MSCs; FPR1^-/-^ -NC, FPR1 knockout mice of normoxia control; FPR1^-/-^ -HC, FPR1 knockout mice of hyperoxia control; FPR1^-/-^ -HM, FPR1 knockout mice of hyperoxia with MSCs. Data are given as mean ± SEM. * P < 0.05 vs. WT-NC. # P < 0.05 vs. WT-HC, % P < 0.05 vs. FPR1^-/-^ -NC.

Lung angiogenesis was quantified by light intensity and the number of vWF positive cells. The fluorescent-immunostained endothelial cell or endothelial cell cluster which was clearly separated from adjacent vessels were considered as a single and countable vessel. Significantly reduced the light intensity and vessel number of vWF positive cells were observed in the WT-HC compared with the NC groups, which were indicatives of impaired angiogenesis, were significantly attenuated with MSCs transplantation in WT group as well as in the FPR1^-/-^ groups regardless of MSC transplantation (Fig 3A). The histological quantification of lung angiogenesis was confirmed by protein level of CD31, which showed similar trend with its histological quantification (Fig 3B). Angiogenic protein marker level, such as vascular endothelial growth factor (VEGF), was also measured in the lung tissues (Fig 3C). The level of VEGF was significantly reduced in the WT-HC group, compared to the WT-NC group, but not reduced in WT-HM. However, in FPR1^-/-^ mice, the VEGF level was not significantly reduced after hyperoxia exposure regardless of MSC transplantation.

### Cell death

**Fig 4:**
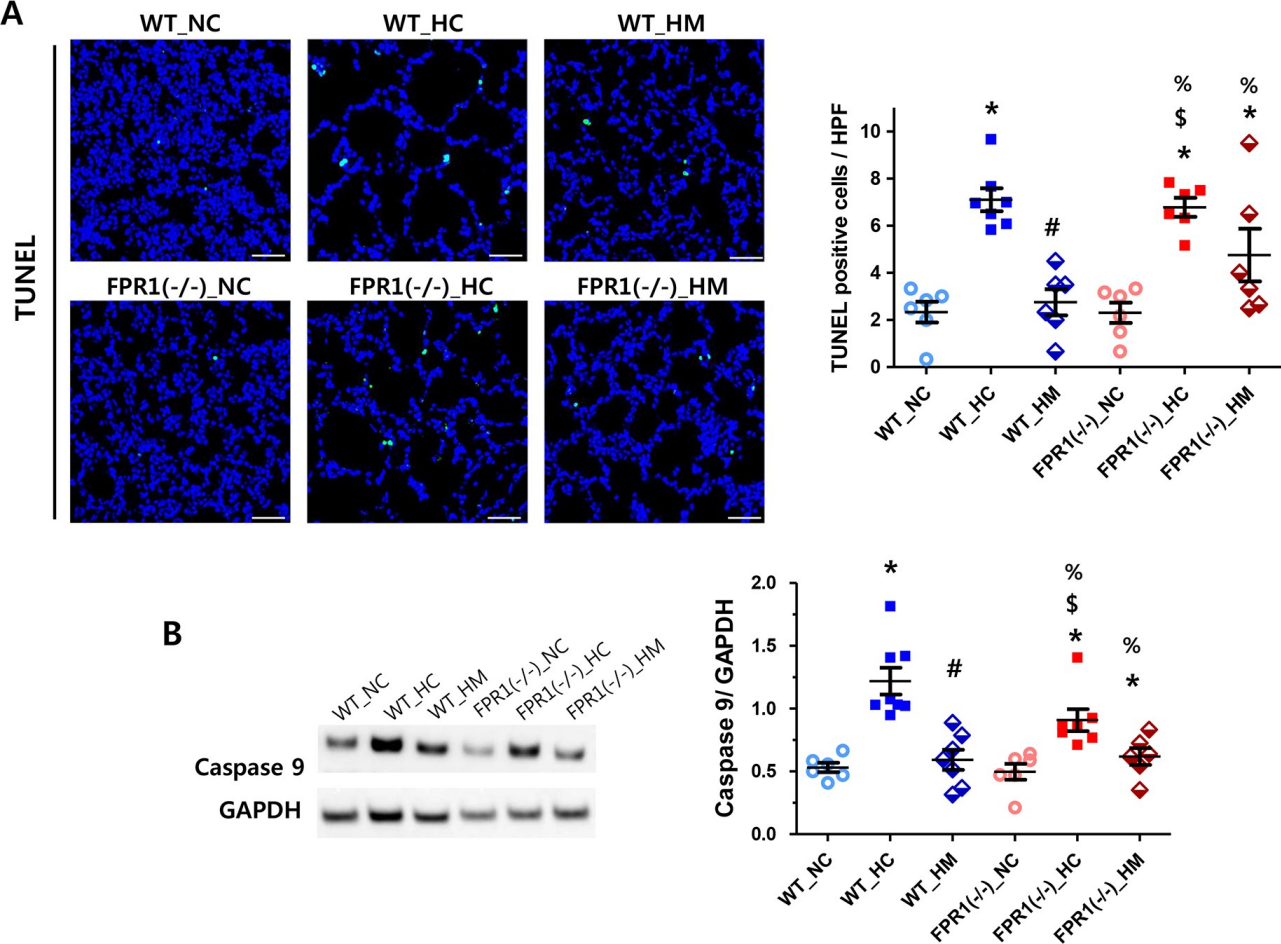
TUNEL-positive apoptotic cells in lung tissue. (A) Representative confocal images of lung tissues stained with TUNEL (green) with DAPI (blue) (left panel, original magnification; ×200, scale bar; 100μm). The number of TUNEL-positive cells per high power field of each group (right panel). (n = 8, 8, 6, 8, 6 and 8 in WT-NC, WT-HC, WT-HM, FPR1^-/-^ -NC, FPR1^-/-^ -HC and FPR1^-/-^ -HM, respectively) (B) Representative western blots of caspase 9 and glyceraldehyde-3-phos phosphatedehydrogenase (GAPDH; loading control) and densitometric analysis of caspase 9 levels normalized to GAPDH (n = 6, 8, 7, 6, 7 and 6 in WT-NC, WT-HC, WT-HM, FPR1^-/-^ -NC, FPR1^-/-^ -HC and FPR1^-/-^ -HM, respectively) WT-NC, wild type mice of normoxia control; WT-HC, wild type mice of hyperoxia control; WT-HM, wild type mice of hyperoxia with MSCs; FPR1^-/-^ -NC, FPR1 knockout mice of normoxia control; FPR1^-/-^ -HC, FPR1 knockout mice of hyperoxia control; FPR1^-/-^ -HM, FPR1 knockout mice of hyperoxia with MSCs. Data are given as mean ± SEM. * P < 0.05 vs. WT-NC. # P < 0.05 vs. WT-HC, $ P < 0.05 vs. WT-HM, % P < 0.05 vs. FPR1^-/-^ -NC.

The number of TUNEL positive cells per high power field was significantly higher in the WT-HC group compared to the NC groups (Fig 4A). The hyperoxia-induced increase in the number of TUNEL-positive cells was significantly attenuated with MSC transplantation in WT group, but not in the FPR1^-/-^ group. Level of total caspase 9 protein, used as a marker for cell death [24], showed similar trend with the number of TUNEL positive cells (Fig 4B).

### Alveolar macrophage and leukocyte infiltration

**Fig 5:**
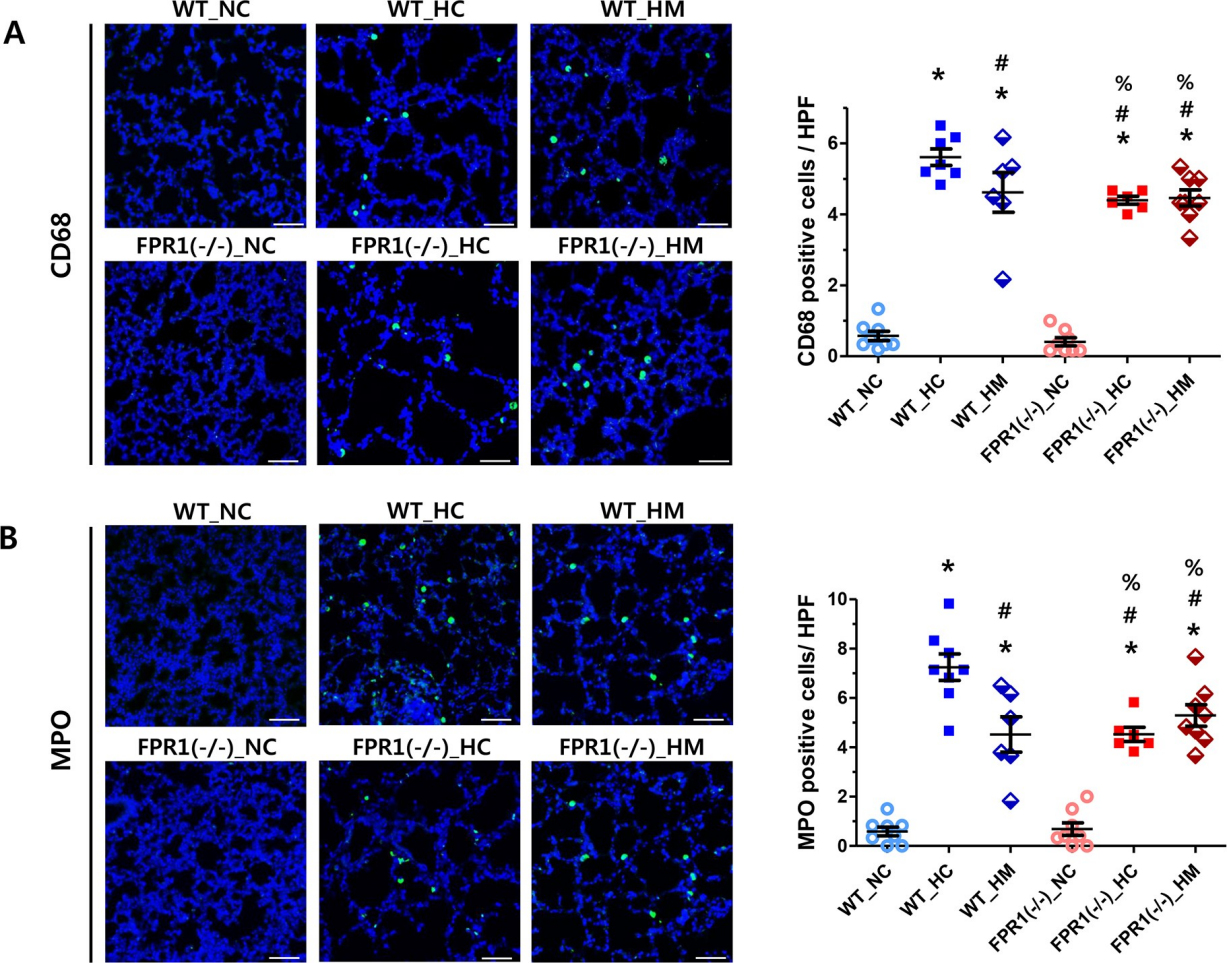
Macrophage and neutrophil infiltrations in lung tissues. (A) Representative confocal images of CD68 (green) with DAPI (blue) (original magnification; ×200, scale bar; 100μm) and the number of CD68-positive cells per high power field of each group (n = 8, 8, 6, 8, 6 and 8 in WT-NC, WT-HC, WT-HM, FPR1^-/-^ -NC, FPR1^-/-^ -HC and FPR1^-/-^ -HM, respectively). (B) Representative confocal images of myeloperoxidase (MPO; green) positive cells with DAPI (blue) and the number of MPO-positive cells per high power field (n = 8, 8, 6, 8, 6 and 8 in WT-NC, WT-HC, WT-HM, FPR1^-/-^ -NC, FPR1^-/-^ -HC and FPR1^-/-^ -HM, respectively). WT-NC, wild type mice of normoxia control; WT-HC, wild type mice of hyperoxia control; WT-HM, wild type mice of hyperoxia with MSCs; FPR1^-/-^ -NC, FPR1 knockout mice of normoxia control; FPR1^-/-^ -HC, FPR1 knockout mice of hyperoxia control; FPR1^-/-^ -HM, FPR1 knockout mice of hyperoxia with MSCs. Data are given as mean ± SEM. * P <0.05 vs. WT-NC. # P < 0.05 vs. WT-HC, % P < 0.05 vs. FPR1^-/-^ -NC.

The numbers of CD68- and MPO-positive cells, indicative of alveolar macrophages and polymorphonuclear neutrophils respectively, were significantly increased in the WT-HC group compared to the NC groups (Fig 5). The hyperoxia induced increase in CD68 and MPO positive cells were significantly attenuated with MSCs transplantation in the WT group, as well as in the FPR1^-/-^ group regardless of MSC transplantation.

## Discussion

In the present study, we have demonstrated that FPR1 is upregulated in the neonatal hyperoxic lung injury showing an increase in lung leukocytes and alveolar macrophages, and ensuing impaired alveolarization and angiogenesis in the WT newborn mice. In addition, a significant attenuation of the hyperoxia-induced lung inflammation and the resultant lung injuries were observed in the WT mice with MSC transplantation with showing significant downregulation of FPR1 levels. Furthermore, FPR1^-/-^ protects hyperoxia induced lung injury regardless of MSC transplantation with similar degree observed in hyperoxic WT neonatal mice with MSC transplantation with showing improved alveolarization and angiogenesis and decreased neutrophil and macrophage infiltration except not showing reduced apoptosis. Therefore, these strongly suggest intratracheal transplantation of MSCs attenuates hyperoxic lung injuries by downregulating, but probably not direct inhibiting FPR1 levels in newborn mice.

Recent studies have suggest that FPR1 signaling, activated by formyl peptides of bacterial and mitochondrial origin, guides neutrophils and macrophages to infectious and sterile sites of inflammation where they contribute to wound healing, but may cause tissue damage when activated excessively [25]. However, the role of FPR1 in the pathogenesis of BPD and in the protective effects of stem cell therapy against BPD has not been fully elucidated yet. Developing a proper animal model, that can simulate clinical BPD of human preterm infants, might be essential to evaluate the pathophysiology and to test possibility of therapeutic applications. We have used prolonged hyperoxia exposure in neonatal mice from birth to P14 in the present study. Neonatal mice survived from prolonged hyperoxia demonstrate significantly impaired alveologenesis and reduced angiogenesis in their lung [26], which closely mimic the histology demonstrated in the lung of human BPD [27]. Neonatal mouse has saccular staged lung development till P5 which corresponds between 26 and 36 weeks of gestation in human [28]. However, there are some limitations for the direct extrapolation of this model to human BPD because neonatal mouse lung with prolonged hyperoxia exposure till P14, almost to the completion of alveolarization might be evolved to chronic hyperoxic lung injury rather than BPD.

Because exact cell type related to FPR1 expression was not evaluated in this study, it is not clear and needs to be clarified whether the increased FPT1 expression in hyperoxic lung resulted from the increase due to more FPR1 expressing cells or each cell expressing more FPR1.

It is well known that MSCs have immunosuppressive capacity through modulating multiple components of the innate immune systems [29, 30] as well as direct inhibition of T-cell function [31] and suppression of B-cell proliferation and function [32]. MSCs can be transplanted between HLA-incompatible individuals because they barley elicit alloreactive lymphocyte proliferative responses [33]. In addition, in our previous studies, the xenotransplantation of MSCs were successfully done to wild-type immunocompetent newborn rats with showing no effect in normal lung [16] and beneficial effects in hyperoxic lung injury [16, 17, 20, 34] same as in our present study.

In concordance with our data, FPR1^-/-^ mice showed reduced lung neutrophil and macrophage infiltration, significantly attenuated endotoxin-induced lung injury [35], and cigarette smoke-induced emphysematous changes [36]. Moreover, acute sterile lung inflammation and injuries induced by mitochondrial formyl peptides and hydrochloric acid were significantly attenuated in the FPR1^-/-^ mice or by a pharmacological FPR1 antagonist [14]. Overall, these findings suggest that activation of FPR1 signaling is one of key drivers of hyperoxia induced lung inflammation and the ensuing lung injuries, thereby highlighting a potential new therapeutic target for BPD.

The precise mechanisms of how MSC transplantation downregulates the level of FPR1 have not been delineated yet. In the present study, FPR1 levels were downregulated but not abrogated with MSC transplantation. In the present study, we just showed that firstly, intratracheal transplantation of MSCs moderately decreased FPR1 expression along with attenuating hyperoxic lung injury and secondly, FPR1 knockout can protect hyerpoxic lung injury also. Here, we did not show the direct evidence that FPR1 is directly involved in the attenuating effect of MSC including data such as proving that overactivation of FPR 1 can inhibit the beneficial effect of MSCs in this hyperoxic lung injury. However, the finding that anti-apoptotic effects were only observed in mice with the MSC transplantation, not in FPR1 FPR1^-/-^ mice may suggest that intratracheal transplantation of MSCs might down-regulate the FPR1 by indirect way through decreased production of mitochondrial formyl peptide or FPR1 activator via protection for cell death in hyperoxic lung injures in the present study. Elevated mitochondrial formyl peptides were observed in the ARDS patients [14], and these peptides drove chemotaxis and activation of neutrophils via FPR1 dependent mechanisms. Collectively, these findings suggest that the downregulation of FPR1 activation observed with MSC transplantation might be attributable to the significant reduction of the upstream FPR1 activator, the mitochondrial formyl peptides, but not to direct inhibition of FPR1 activation. Further studies will be necessary to clarify this.

While FPR1 is a dominant pro-inflammatory formyl peptide receptor [37], the role of FPR2 is promiscuous with ligand dependent pro-inflammatory or anti-inflammatory effects [38]. We preliminarily observed significant downregulation of FPR2 along with hyperoxia-induced lung inflammation and injuries (unpublished data). However the precise role of FPR2 in hyperoxia-induced lung inflammation and injuries still remains unclear. Activation of FPR2 has been shown to induce both pro-inflammatory [39] and anti-inflammatory signals [40] depending on the character of ligands [41]. Therefore, as a next step, we plan to conduct experiments to elucidate the role of FPR2 in mediating this hyperoxia-induced lung injury as well as the protective effects of MSCs including the expression of FPR2 in the present animal groups of mice FPR1^-/-^ or FPR2^-/-^ mice modeling system.

In this study, the anti-apoptotic effects were observed only in mice with the MSC transplantation, not in FPR1^-/-^ mice. This might be possible because hyperoxia induced cell death and inflammation occur via different distinct pathways. These findings suggest that while FPR1 plays a critical role as a regulator of the inflammatory process and the resultant lung injuries in BPD, the anti-apoptotic effects of MSC transplantation were mediated by secretion of other paracrine factors and signaling pathways [42]. For more exact explanation for this, further study is needed to investigate whether this finding is associated with increases in ongoing cell proliferation or not.

The present study has several limitations. First, we did not measure the level of formyl peptide, a main ligand of FPR1, which acts as a damage-associated molecular pattern [43]. probably also in hyperoxic lung injury. Therefore, further study is needed to investigate whether the increased level of formyl peptide has direct link with the increased level of FPR1 expression in hyperoxic lung. Second, we did not clarify the exact mechanism how MSC transplantation attenuated hyperoxic lung injury via moderately reducing the FPR1 expression level. Thus, further studies are also needed to know whether its’ mechanism might be direct by showing inhibiting effect of overexpression of FPR1 on the attenuating hyperoxic lung injury by MSC transplantation or indirect by demonstrating indirect effects on reduced FPR activation through decreased formyl peptide production via paracrine effects of MSCs transplanted in hyperoxic lung. On the other hand, the present study also gives us the future perspectives regarding possible development of pharmacological FPR-1 inhibitors in the BPD in which the inflammation plays a pivotal role for the disease process.

In summary, the increased lung infiltration of neutrophils and alveolar macrophages as well as the ensuing lung injuries, along with significant upregulation of FPR1 levels in the WT hyperoxic newborn mice, suggest the critical role of FPR1 in mediating the hyperoxia-induced lung inflammation and injuries. Significant attenuation of the hyperoxia-induced lung inflammation and injuries with MSC transplantation in WT mice as well as in FPR1^-/-^ mice regardless of MSCs transplantation suggests that FPR1 could be a potential novel therapeutic target in BPD. However, the anti-apoptotic effects observed only with MSC transplantation but not in FPR1^-/-^ mice suggest another paracrine mechanism for protection against BPD.

## Supporting information

S1 Fig mRNA level of formyl peptide receptor (FPR) 1 in lung tissues in normoxic condition. In normoxia condition, the intratracheal transplantation of MSCs did not significantly change the FPR1 mRNA expression level in WT and FPR1 -/ mice, compared to control groups. WT-NC, wild type mice of normoxia control; WT-NM (n = 2), wild type mice of normoxia with MSCs (n = 3); FPR1 -/-NC, FPR1 lacking mice of normoxia control; FPR1 -/-NM (n = 2), FPR1 lacking mice of normoxia with MSCs (n = 3).
(PDF)

S2 Fig Genotyping of FPR1 knockout mice. mRNA of FPR1 knockout mice (430bp band) is distinguished from the wild type mRNA (350bp band) by PCR.
(PDF)
