# Supplementary material for: Intratracheal transplantation of mesenchymal stem cells attenuates hyperoxia-induced lung injury by down-regulating, but not direct inhibiting formyl peptide receptor 1 in the newborn mice
Source: PLoS One. 2018 Oct 24;13(10):e0206311. doi: 10.1371/journal.pone.0206311 (PMC6200259; doi:10.1371/journal.pone.0206311)
Supplement: S1 Fig — In normoxia condition, the intratracheal transplantation of MSCs did not significantly change the FPR1 mRNA expression level in WT and FPR1 -/ mice, compared to control groups. WT-NC, wild type mice of normoxia control; WT-NM (n = 2), wild type mice of normoxia with MSCs (n = 3); FPR1 -/-NC, FPR1 lacking mice of normoxia control; FPR1 -/-NM (n = 2), FPR1 lacking mice of normoxia with MSCs (n = 3). (PDF) [file pone.0206311.s001.pdf]

Supporting Information

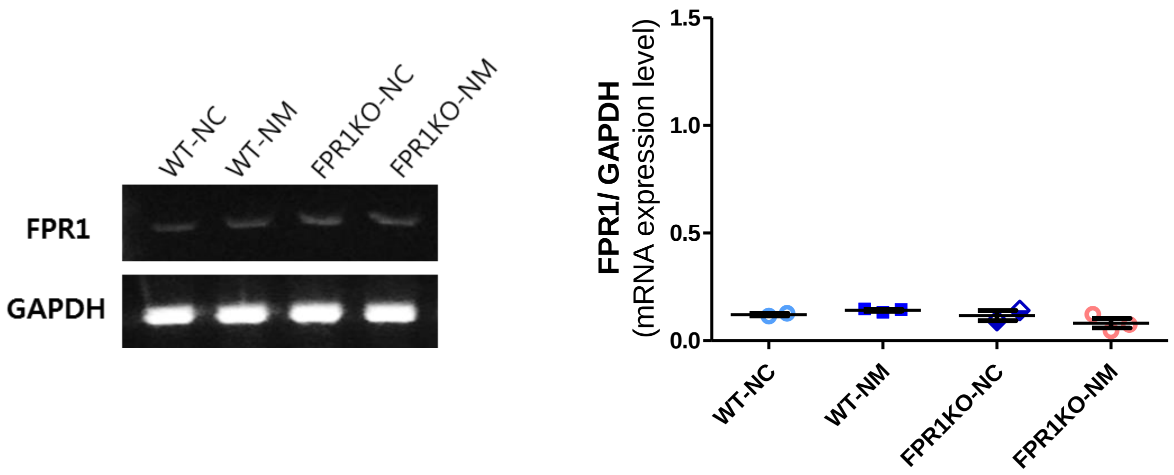

S1 Fig. RNA level of formyl peptide receptor (FPR) 1 in lung tissues in normoxic condition. In normoxia condition, the intratracheal transplantation of MSCs did not significantly change the FPR1 mRNA expression level in WT and FPR1$^{-/-}$ mice, compared to control groups. WT-NC, wild type mice of normoxia control; WT-NM (n=2), wild type mice of normoxia with MSCs (n=3); FPR1$^{-/-}$-NC, FPR1 lacking mice of normoxia control; FPR1$^{-/-}$-NM (n=2), FPR1 lacking mice of normoxia with MSCs (n=3).
