# Supplementary material for: Intratracheal transplantation of mesenchymal stem cells attenuates hyperoxia-induced lung injury by down-regulating, but not direct inhibiting formyl peptide receptor 1 in the newborn mice
Source: PLoS One. 2018 Oct 24;13(10):e0206311. doi: 10.1371/journal.pone.0206311 (PMC6200259; doi:10.1371/journal.pone.0206311)
Supplement: S2 Fig — mRNA of FPR1 knockout mice (430bp band) is distinguished from the wild type mRNA (350bp band) by PCR. (PDF) [file pone.0206311.s002.pdf]

Supporting Information

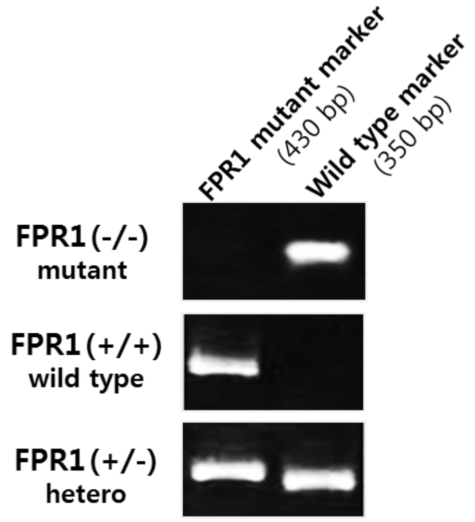

S2 Fig. Genotyping of FPR1 mutant mice. Mutated mRNA of FPR1 (430bp band) is distinguished from the wild type mRNA (350bp band) by PCR.
